# Depression and anxiety among diabetics in primary care : a cross-sectional study

**DOI:** 10.1192/j.eurpsy.2021.634

**Published:** 2021-08-13

**Authors:** R. Gniwa Omezzine, W. Bouali, A. Belguith Sriha, L. Zarrouk

**Affiliations:** 1 Department Of Family Medicine. Tunisia., Monastir Faculty of Medicine, Mahdia, Tunisia; 2 Department Of Psychiatry, University Hospital Of Mahdia, Tunisia., Psychiatry, Mahdia, Tunisia; 3 Department Of Community Medicine, Monastir Faculty of Medicine, Mahdia, Tunisia

**Keywords:** diabetes, anxiety, depression, cross-sectional study

## Abstract

**Introduction:**

Diabetes mellitus is one of the most frequent chronic diseases in Tunisia. Individuals with diabetes mellitus may have concurrent mental health disorders and are shown to have poorer disease outcomes.

**Objectives:**

The aim of this study was to determine the prevalence of depression and anxiety in diabetics attending the primary care setting.

**Methods:**

This was a cross-sectional survey carried out over two months and including diabetic patients followed up at the consultation for chronic diseases at the primary care center of Hiboun, in Mahdia, Tunisia. The validated Hosiptal Anxiety and Depression scale (HAD) questionnaire was used as a screening tool for the symptoms of depression and anxiety.

**Results:**

A total of 64 patients (24 men and 40 women) was enrolled. The average age was 54.5 ± 7.2 years. The mean duration of diabetes was 8.2 ± 2.3 years. The average HbA1c level was 9.1%.Over 48% of patients were overweight. The prevalence of Depression and anxiety among patients with diabetes from our study was 29.6% and 40.6%, respectively. Depression was found to be significantly associated with marital status of widowed, HbA1c level of more than 8.5%, and a family history of psychiatric illness. anxiety was significantly associated with females, unmployement and HbA1c level of more than 8.5%.

**Conclusions:**

Screening of high risk Type II diabetics for depression and anxiety symptoms in the primary care setting is recommended at regular intervals.

